# Arterial and venous thromboembolic events in patients with cancer treated with targeted therapies: a population-based cohort study

**DOI:** 10.1016/j.eclinm.2025.103440

**Published:** 2025-08-21

**Authors:** Florian Moik, Erzsébet Horváth-Puhó, Cihan Ay, Ingrid Pabinger, Frits Mulder, Nick van Es, Henrik Toft Sørensen

**Affiliations:** a Division of Oncology, Department of Internal Medicine, Medical University of Graz, Graz, Austria; b Clinical Division of Hematology and Hemostaseology, Department of Medicine I, Comprehensive Cancer Center Vienna, Medical University of Vienna, Vienna, Austria; c Department of Clinical Epidemiology and Center for Population Medicine, Aarhus University Hospital and Aarhus University, Aarhus, Denmark; d Department of Vascular Medicine, Amsterdam Cardiovascular Sciences, Amsterdam University Medical Center, University of Amsterdam, Amsterdam, the Netherlands

**Keywords:** Cancer, Targeted therapy, Venous thromboembolism, Arterial thromboembolic event, Cohort study

## Abstract

**Background:**

Emerging data suggest a substantial risk of arterial and venous thromboembolic events (ATE/VTE) associated with targeted cancer therapies. We examined the association between selected targeted therapies and ATE/VTE-risk using Danish population-based healthcare data.

**Methods:**

We identified 41,744 patients with cancer treated with selected targeted therapies between January 2004 and December 2020. We computed cumulative incidence functions and 95% confidence intervals (CIs) of ATE/VTE after therapy initiation, considering death as competing event. A multivariable Cox proportional hazards regression analysis with time-varying exposure to targeted therapy was conducted for selected cancers, calculating hazard ratios (HRs) and 95% CIs for ATE/VTE, enabling the comparison of the time periods with and without targeted therapy, adjusting for age, sex, comorbidity burden, cancer stage, and year of diagnosis.

**Findings:**

The three-year cumulative ATE-incidence was 3.7% (95% CI: 3.2–4.2) with immune checkpoint inhibitors (ICI; n = 7880), 3.4% (95% CI: 2.8–4.1) with multi-kinase inhibitors (MKI; n = 3394), 2.6% (95% CI: 1.9–3.5) with cyclin dependent kinase (CDK) 4/6-inhibitors (n = 1966), 2.5% (95% CI: 1.0–5.4) with anaplastic lymphoma kinase (ALK)-/ROS1-targeted therapies (n = 199), 2.6% (95% CI: 2.2–2.9) with epidermal growth factor receptor (EGFR)-targeted therapies (n = 8603), 2.4% (95% CI: 2.1–2.7) with vascular endothelial growth factor (VEGF)-targeted therapies (n = 12,802), and 1.4% (95% CI: 1.2–1.6) with human epidermal growth factor receptor 2 (HER2)-targeted therapies (n = 11,683). The three-year VTE-incidence was highest for EGFR- (9.3% [95% CI: 8.7–9.9]), ALK/ROS- (9.2% [95% CI: 5.7–13.8]), VEGF-targeted therapies (8.8% [95% CI: 8.3–9.3]), and ICI (8.1% [95% CI: 7.5–8.8]), followed by 7.5% (95% CI: 6.7–8.5) with MKI, 6.9% (95% CI: 5.7–8.3) with CDK4/6-inhibitors, and 3.4% (95% CI: 3.1–3.8) with HER2-targeted therapies. Among patients with selected cancer types, time-dependent exposure to certain targeted therapies was associated with an increased risk of ATE and/or VTE.

**Interpretation:**

Selected targeted therapies pose a clinically meaningful risk of ATE and VTE in patients with cancer.

**Funding:**

Department of Clinical Epidemiology, Center for Population Medicine, 10.13039/100007605Aarhus University and 10.13039/100007606Aarhus University Hospital, Denmark and the 10.13039/501100011958Independent Research Fund Denmark (3101-00102B).

**Research in context**

**Evidence before this study**

Patients with cancer are at an increased risk of arterial and venous thromboembolism, contributing to mortality, morbidity, and delay in cancer treatments. An increasing proportion of patients with cancer is treated with targeted therapies, yet limited data is available on the risk of thromboembolic events under different targeted therapies from clinical trials. Emerging data from observational studies suggest a high risk of thromboembolic events for certain targeted agent groups. Detailed data on the risk profiles of thromboembolic events in patients treated with targeted cancer therapies are needed to guide clinical management.

**Added value of this study**

Based on Danish population-based data, we provide a comprehensive analysis of the risk of thromboembolic events in patients with cancer treated with different targeted therapies. We found a considerable risk of arterial and venous thromboembolic events for selected targeted therapies, varying across treatment and cancer types.

**Implications of all the available evidence**

The development of treatment-specific surveillance and prevention strategies for arterial and venous thromboembolic events in patients with cancer treated with targeted therapies is warranted.

## Introduction

Patients with cancer have elevated risks of arterial and venous thromboembolic events (ATE and VTE),^1^^,^^2^ leading to increased morbidity, cancer-therapy delays, and mortality.^1^, ^2^, ^3^, ^4^ The risk of ATE and VTE among patients with cancer varies considerably and depends on patient-specific and cancer-specific factors.^5^, ^6^, ^7^ Cancer treatments, such as surgery and radiotherapy along with systemic therapies like chemotherapy and hormonal treatments, further affect the risk of ATE and VTE.^5^^,^^6^^,^^8^

The therapeutic landscape in oncology is evolving and molecularly targeted therapies and immunotherapies are increasingly being used.^9^^,^^10^ Limited data are available regarding the risk of thromboembolic events after the initiation of targeted therapies^3^^,^^11^ but prothrombotic effects have been established for selected groups of therapies (e.g., anti-angiogenic therapy).^12^, ^13^, ^14^ For other therapies, the thromboembolic risk profiles are less clear. Data from clinical trials are limited, partly as a result of commonly used frequency and severity thresholds in the reporting of adverse events.^15^, ^16^, ^17^ Observational studies have reported a high risk of thromboembolic events for several targeted therapies used in clinical practice, but these studies were limited in sample size and prone to selection bias.^18^, ^19^, ^20^, ^21^ Therefore, comprehensive analysis of the thromboembolic risk associated with targeted cancer therapies remains an unresolved clinical need.

Using population-based data from Danish medical and administrative registries, we conducted a cohort study to examine the risk of ATE and VTE in patients with cancer treated with immune checkpoint inhibitors (ICI), multi-kinase inhibitors (MKI), and agents targeting epidermal growth factor receptors (EGFR), vascular endothelial growth factor (VEGF), human epidermal growth factor receptor 2 (HER2), anaplastic lymphoma kinase (ALK) or ROS1, or cyclin dependent kinase 4/6 (CDK4/6).

## Methods

### Study design

For this population-based cohort study, we used nationwide data recorded in the Danish registries from January 1, 2004 through December 31, 2020. Denmark has a universal tax-funded healthcare system with free access to general practitioners and hospitals.^22^^,^^23^ Individual-level data from all Danish registries can be linked via a unique personal identification number assigned at birth or upon immigration and registered in the Danish Civil Registration System (DCRS).^23^

We used the Danish Cancer Registry (DCR)^24^ to identify all patients with a new cancer diagnosis during the study period. Patients with cancer treated with targeted therapies were identified from the Danish National Patient Registry (DNPR) using procedure codes and the Danish National Prescription Registry (NPR) using *Anatomical Therapeutic Chemical* (ATC) codes.^25^, ^26^, ^27^ A description of the registries and a complete list of the treatment and diagnosis codes used in this study can be found in the Supplemental Tables S1–S3.

### Ethics

The study was reported to the Danish Data Protection Agency (record number 2016-051-000001-811). Ethical approval and informed consent are not required for register-based studies in Denmark.

### Study cohorts

**Fig. 1:**
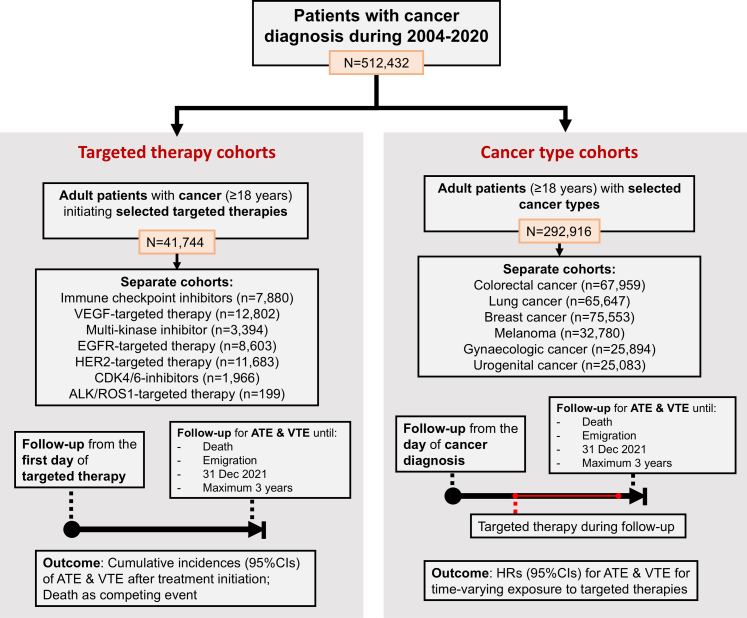
**Study flowchart illustrating the study cohorts.** Abbreviations: 95% CIs: 95% confidence intervals, ALK/ROS1: Anaplastic lymphoma kinase or ROS1, ATE: arterial thromboembolic event, CDK4/6: cyclin-dependent kinase 4/6, EGFR: epidermal growth factor receptor, HER-2: human epidermal growth factor receptor 2, HRs: hazard ratios, VEGF: vascular endothelial growth factor, VTE: venous thromboembolism.

Based on the identified patients with cancer diagnosis in the study period, we created two types of study cohorts according to type of targeted therapy and type of cancer (Fig. 1).

#### Targeted therapy cohorts

First, we established seven cohorts of patients with cancer treated with selected targeted therapies, i.e., ICI, MKI, and therapies targeted at VEGF, HER2, EGFR, CDK4/6, or ALK-/ROS. The targeted therapies were selected based on prior data regarding the risk of ATE and VTE and the availability of sufficient numbers of treated patients (i.e., ≥100 treated patients).^3^^,^^11^ The cohorts could potentially overlap because patients could have received different targeted therapies throughout the course of therapy.

#### Cancer-type cohorts

Second, from all patients with a new diagnosis of cancer during the study period, we defined six cancer-type cohorts: lung cancer, colorectal cancer, melanoma, urogenital cancer, breast cancer, and gynecological cancer cohorts.

### Study outcomes

The study outcomes were (i) ATE, covering myocardial infarction, ischemic stroke, and peripheral arterial occlusion, and (ii) VTE, covering deep vein thrombosis, pulmonary embolism, and other venous thromboses. Data on study outcomes were retrieved from the DNPR (Supplemental Tables S1 and S3). Unspecified stroke diagnosis was included in the ischemic stroke group, because most unclassified events are of ischemic etiology.^28^

### Covariates and other variables

Patient characteristics were obtained from the DCRS (date of birth and sex), and from the DCR (cancer type, localized/regional/metastatic stage, and year of cancer diagnosis). Information on comorbidities was obtained from the DNPR according to the Charlson Comorbidity Index score, which includes 19 major medical conditions (Supplemental Table S3).^29^^,^^30^

### Statistics

#### Targeted therapy cohorts

Patients were followed from the initiation of the analyzed targeted therapy until an outcome of interest, death, emigration, December 31, 2021, or for a maximum follow-up of three years, whichever came first. Six-month, one-year-, two-year, and three-year cumulative incidences of ATE and VTE (overall and for selected cancer subgroups) were computed using nonparametric estimation of cumulative incidence functions and corresponding 95% confidence intervals (CI), accounting for all-cause mortality as a competing event.^31^^,^^32^

#### Cancer-type cohorts

Patients in the cancer-type cohorts were followed from the cancer diagnosis date until an outcome of interest, death, emigration, December 31, 2021, or for a maximum of three years after cancer diagnosis, whichever came first. Cox proportional hazards regression analysis was used to compute hazard ratios (HRs) and corresponding 95% CIs for time-dependent exposure to targeted therapies, adjusted for patient age, sex, Charlson Comorbidity Index score, cancer stage, and year of cancer diagnosis, all at cancer diagnosis. Patients' exposure status was updated at targeted therapy initiation, allowing for a comparison of unexposed and therapy-exposed periods within distinct cancer type cohorts, with each treatment group analyzed separately.

All analyses were conducted using SAS version 9.4 (SAS Institute, INC, Cary, NC, USA) and R (version: RStudio/2023.09.1+494) for data visualization.

### Role of funding source

This study was supported by the Department of Clinical Epidemiology, Center for Population Medicine, Aarhus University and Aarhus University Hospital, Denmark and by the Independent Research Fund Denmark (3101-00102B). The Department of Clinical Epidemiology is involved in studies with funding for various companies as research grants to (and administered) Aarhus University. None of these studies have any relation to the present study and the Funders no role in study design, data collection, data analyses, interpretation, or writing of report.

## Results

During the study period, 512,432 patients were diagnosed with cancer.

### Targeted therapy cohorts: cumulative incidence of ATE and VTE

Overall, we identified 41,744 patients with cancer who initiated one of the predefined targeted therapies during the study period. In 26,279 patients (62.9%), targeted therapy was initiated within one year after cancer diagnosis, while 20,403 (48.9%) started targeted therapy within six months. Patient characteristics of the different targeted therapy cohorts are provided in Table 1 and Supplemental Tables S4–S6. Overall, 11.2% of patients (n = 4659) were included in ≥1 targeted therapy group (Supplemental Table S7).

**Table 1:** Targeted therapy cohorts: patient characteristics.

	Targeted therapy	ICI	VEGF-targeted	MKI	HER2-targeted	EGFR-targeted	ALK/ROS1-targeted	CDK4/6-targeted
Overall N	**N = 41,744**	**N = 7880**	**N = 12,802**	**N = 3394**	**N = 11,683**	**N = 8603**	**N = 199**	**N = 1966**
Age (median, IQR)^a^	65 (56–72)	68 (60–74)	66 (58–73)	67 (59–73)	59 (50–68)	66 (58–72)	65 (54–72)	69 (58–75)
Female (n, %)	26,068 (62.4%)	3565 (45.2%)	6798 (53.1%)	1105 (32.6%)	10,862 (93.0%)	4116 (47.8%)	118 (59.3%)	1943 (98.8%)
Cancer stage (n, %)^b^								
Localized	8113 (19.4%)	916 (11.6%)	1941 (15.2%)	356 (10.5%)	4260 (36.5%)	607 (7.1%)	15 (7.5%)	480 (24.4%)
Regional	12,148 (29.1%)	2309 (29.3%)	2941 (23.0%)	750 (22.1%)	4412 (37.8%)	2207 (25.7%)	29 (14.6%)	805 (40.9%)
Metastatic	12,855 (30.8%)	2548 (32.3%)	5809 (45.4%)	1193 (35.2%)	1030 (8.8%)	4201 (48.8%)	131 (65.8%)	203 (10.3%)
CCI score (n, %)^a^								
0	28,463 (68.2%)	4706 (59.7%)	8907 (69.6%)	1763 (51.9%)	9203 (78.8%)	5665 (65.8%)	134 (67.3%)	1368 (69.6%)
1	8328 (20.0%)	1867 (23.7%)	2620 (20.5%)	748 (22.0%)	1779 (15.2%)	1863 (21.7%)	39 (19.6%)	399 (20.3%)
≥2	4953 (11.9%)	1307 (16.6%)	1275 (10.0%)	883 (26.0%)	701 (6.0%)	1075 (12.5%)	26 (13.1%)	199 (10.1%)
Year of treatment (n, %)								
2004–2009	5779 (13.8%)	–	1715 (13.4%)	–	3148 (26.9%)	1323 (15.4%)	–	–
2010–2015	15,579 (37.3%)	655 (8.3%)	6124 (47.8%)	1688 (49.7%)	4529 (38.8%)	4740 (55.1%)	66 (33.2%)	–
2016–2020	20,386 (48.8%)	7225 (91.7%)	4963 (38.8%)	1706 (50.3%)	4006 (34.3%)	2540 (29.5%)	133 (66.8%)	1966 (100%)
Time to targeted therapy, months (median, IQR)^c^	6.4 (2.0–22.6)	10.1 (2.0–28.3)	8.4 (1.9–22.7)	11.0 (2.2–36.7)	4.2 (3–8.8)	12.1 (2.4–27.4)	3.2 (0.8–17.2)	63.7 (16.0–129.2)

Abbreviations: ALK: anaplastic lymphoma kinase, ATE: arterial thromboembolic events, CCI: Charlson Comorbidity Index, CDK: cyclin dependent kinase, EGFR: epidermal growth factor receptor, HER2: human epidermal growth factor receptor 2, ICI: immune checkpoint inhibitors, MKI: multi-kinase inhibitors, VEGF: vascular endothelial growth factor, VTE: venous thromboembolism.

a At therapy initiation.

b At cancer diagnosis.

c Time from cancer diagnosis to targeted therapy.

#### Immune checkpoint inhibitors

In total, 7879 patients were treated with ICI, primarily for lung cancer (n = 3368; 43%), melanoma (n = 1855; 24%), and urogenital cancer (n = 917; 12%). Over a median follow-up of 15.5 months (interquartile range [IQR]: 6.8–26.8), ATE occurred in 245 patients (3.1%) and VTE occurred in 591 patients (7.5%).

**Fig. 2:**
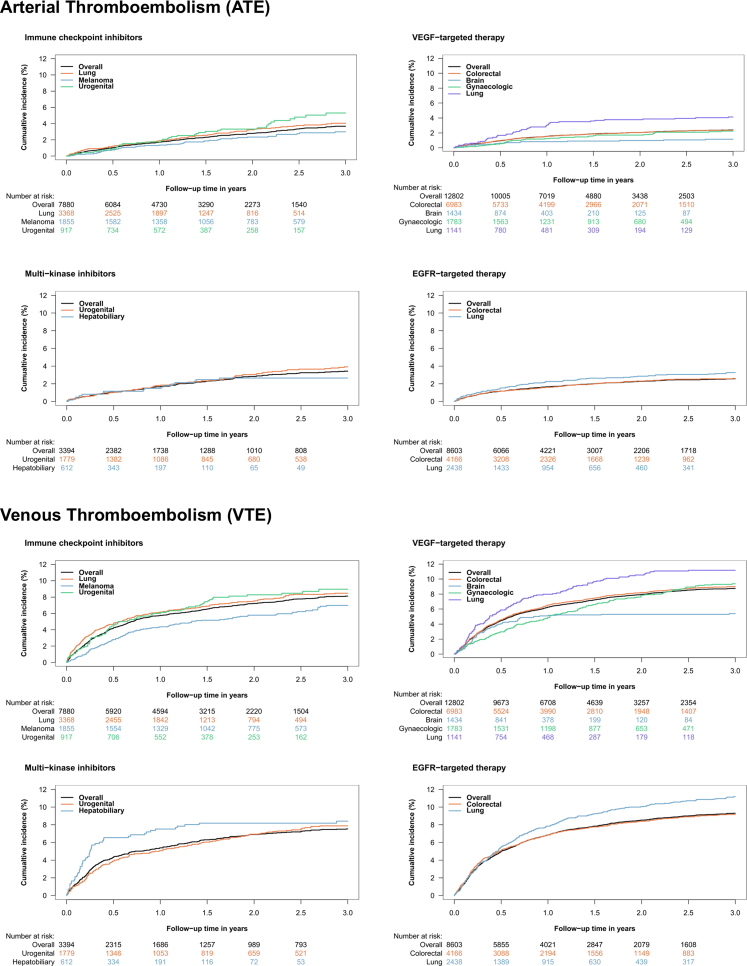
**Targeted therapy cohorts: cumulative incidence of ATE and VTE.** Displayed cumulative incidence values include overall estimates and cumulative risk estimates in the largest subgroups according to the underlying cancer types. Abbreviations: EGFR: epidermal growth factor receptor, VEGF: vascular endothelial growth factor.

The cumulative incidence of ATE three years after ICI initiation was 3.7% (95% CI: 3.2–4.2) (Fig. 2, Table 2). We observed similar cumulative incidences of ATE when the cohort was restricted to patients with lung cancer (4.0% [95% CI: 3.3–4.8]), melanoma (3.0% [95% CI: 2.2–3.9]) and urogenital cancer (5.3% [95% CI: 3.8–7.2]). The cumulative three-year incidence of VTE after ICI initiation was 8.1% (7.5–8.8) (Fig. 2, Table 2). Similar cumulative incidences were observed in patients with lung cancer (8.5% [95% CI: 7.5–9.5]), melanoma (7.0% [95% CI: 5.8–8.3]), and urogenital cancer (9.0% [95% CI: 7.1–11.1]).

**Table 2:** Targeted therapy cohorts: cumulative incidence of ATE and VTE after therapy initiation, overall and by cancer-type.

Targeted therapy	N	Cumulative incidence of ATE, % (95% CI)				Cumulative incidence of VTE, % (95% CI)			
		6 months	1 year	2 years	3 years	6 months	1 year	2 years	3 years
**Immune checkpoint Inhibitors (ICI)**	**7880**	1.0 (0.8–1.3)	1.7 (1.4–2.0)	2.8 (2.4–3.2)	3.7 (3.2–4.2)	4.2 (3.8–4.7)	5.8 (5.3–6.3)	7.2 (6.7–7.8)	8.1 (7.5–8.8)
Lung	3368	1.2 (0.9–1.6)	1.9 (1.5–2.4)	3.2 (2.7–3.9)	4.0 (3.3–4.8)	4.8 (4.1–5.5)	6.2 (5.4–7.0)	7.5 (6.7–8.5)	8.5 (7.5–9.5)
Melanoma	1855	0.8 (0.4–1.2)	1.4 (0.9–2.0)	2.3 (1.7–3.1)	3.0 (2.2–3.9)	2.8 (2.1–3.6)	4.4 (3.5–5.4)	5.8 (4.8–6.9)	7.0 (5.8–8.3)
Urogenital	917	0.9 (0.4–1.7)	1.7 (1.0–2.8)	3.3 (2.3–4.7)	5.3 (3.8–7.2)	4.5 (3.3–6.0)	6.1 (4.7–7.8)	8.3 (6.6–10.2)	9.0 (7.1–11.1)
**VEGF-targeted**	**12,802**	1.0 (0.8–1.2)	1.5 (1.3–1.8)	2.1 (1.8–2.3)	2.4 (2.1–2.7)	4.5 (4.1–4.9)	6.3 (5.9–6.7)	8.0 (7.5–8.4)	8.8 (8.3–9.3)
Colorectal	6983	1.0 (0.8–1.3)	1.5 (1.3–1.8)	2.1 (1.8–2.4)	2.4 (2.0–2.7)	4.6 (4.1–5.1)	6.5 (6.0–7.1)	8.2 (7.6–8.9)	9.0 (8.3–9.7)
Gynecological	1783	0.6 (0.3–1.0)	1.2 (0.8–1.8)	1.7 (1.2–2.4)	2.3 (1.6–3.0)	3.0 (2.3–3.8)	4.9 (4.0–6.0)	7.8 (6.6–9.1)	9.4 (8.0–10.8)
Brain	1434	0.6 (0.3–1.2)	0.8 (0.5–1.4)	1.0 (0.6–1.6)	1.2 (0.7–1.8)	4.0 (3.1–5.2)	5.1 (4.0–6.3)	5.3 (4.2–6.6)	5.4 (4.3–6.6)
Lung	1141	1.7 (1.0–2.5)	3.1 (2.2–4.2)	3.8 (2.8–5.0)	4.1 (3.1–5.4)	5.9 (4.6–7.3)	8.0 (6.5–9.6)	10.5 (8.8–12.4)	11.2 (9.4–13.1)
**Multi-kinase inhibitors (MKI)**	**3394**	1.0 (0.7–1.4)	1.7 (1.3–2.2)	2.8 (2.3–3.4)	3.4 (2.8–4.1)	4.3 (3.7–5.1)	5.4 (4.7–6.2)	6.9 (6.1–7.8)	7.5 (6.7–8.5)
Urogenital	1779	1.0 (0.6–1.6)	1.9 (1.3–2.6)	3.0 (2.3–3.9)	3.9 (3.1–4.9)	3.9 (3.1–4.9)	5.1 (4.1–6.2)	6.9 (5.8–8.2)	7.9 (6.7–9.2)
Hepatobiliary	612	1.1 (0.5–2.3)	1.5 (0.7–2.7)	2.7 (1.6–4.2)	2.7 (1.6–4.2)	6.5 (4.8–8.7)	7.5 (5.6–9.8)	8.2 (6.2–10.5)	8.4 (6.4–10.8)
**EGFR-targeted**	**8603**	1.2 (1.0–1.4)	1.7 (1.4–2.0)	2.3 (2.0–2.6)	2.6 (2.2–2.9)	5.0 (4.6–5.5)	6.9 (6.3–7.4)	8.5 (7.9–9.1)	9.3 (8.7–9.9)
Colorectal	4166	1.2 (0.9–1.5)	1.6 (1.2–2.0)	2.3 (1.9–2.8)	2.6 (2.1–3.1)	5.1 (4.5–5.8)	6.8 (6.1–7.6)	8.4 (7.6–9.3)	9.2 (8.3–10.1)
Lung	2438	1.5 (1.1–2.1)	2.3 (1.7–2.9)	2.9 (2.3–3.6)	3.3 (2.6–4.0)	5.5 (4.6–6.5)	7.8 (6.8–9.0)	10.1 (8.9–11.3)	11.2 (10.0–12.5)
**HER2-targeted**	**11,683**	0.4 (0.3–0.5)	0.6 (0.5–0.7)	1.0 (0.9–1.2)	1.4 (1.2–1.6)	1.4 (1.2–1.7)	1.9 (1.7–2.2)	2.7 (2.4–3.0)	3.4 (3.1–3.8)
Breast	10,370	0.2 (0.2–0.3)	0.5 (0.3–0.6)	0.9 (0.7–1.1)	1.3 (1.0–1.5)	1.1 (0.9–1.3)	1.5 (1.3–1.7)	2.1 (1.9–2.4)	2.9 (2.6–3.3)
Gastroesophageal	727	1.5 (0.8–2.6)	1.9 (1.1–3.1)	2.4 (1.4–3.7)	2.6 (1.6–4.0)	5.5 (4.0–7.3)	6.9 (5.2–8.9)	8.9 (7.0–11.1)	9.2 (7.3–11.5)
**CDK4/6 inhibitors**	**1966**	0.4 (0.2–0.8)	0.8 (0.5–1.2)	1.7 (1.2–2.4)	2.6 (1.9–3.5)	1.9 (1.4–2.6)	3.3 (2.5–4.1)	5.3 (4.4–6.4)	6.9 (5.7–8.3)
Breast	1780	0.3 (0.1–0.6)	0.6 (0.3–1.1)	1.4 (0.9–2.0)	2.3 (1.6–3.2)	2.0 (1.4–2.7)	3.3 (2.6–4.2)	5.3 (4.3–6.5)	7.1 (5.9–8.5)
**ALK/ROS-targeted**	**199**	1.0 (0.2–3.3)	2.5 (1.0–5.4)	2.5 (1.0–5.4)	2.5 (1.0–5.4)	6.0 (3.3–9.9)	7.0 (4.0–11.1)	8.1 (4.8–12.4)	9.2 (5.7–13.8)
Lung	180	1.1 (0.2–3.6)	2.8 (1.1–6.0)	2.8 (1.1–6.0)	2.8 (1.1–6.0)	5.0 (2.5–8.9)	6.1 (3.2–10.3)	7.3 (4.1–11.7)	8.5 (5.0–13.2)

Abbreviations: ALK: anaplastic lymphoma kinase, ATE: arterial thromboembolic events, CDK: cyclin dependent kinase, EGFR: epidermal growth factor receptor, HER2: human epidermal growth factor receptor 2, ICI: immune checkpoint inhibitors, MKI: multi-kinase inhibitors, VEGF: vascular endothelial growth factor, VTE: venous thromboembolism.

Bold values indicate the treatment subgroup, whereas the following rows represent subgroups of different cancer types within the respective targeted treatment cohort.

#### VEGF-targeted therapy

VEGF-targeted therapies were used in 12,802 patients, primarily for colorectal cancer (n = 6983; 55%), gynecological cancer (n = 1783; 14%), brain cancer (n = 1434; 11.2%), and lung cancer (n = 1141; 9%). During a median follow-up of 13.7 months (IQR: 6.8–25.7), 297 patients had an ATE (2.3%) and 1104 had a VTE (8.6%).

The cumulative three-year incidence of ATE was 2.4% (95% CI: 2.1–2.7). The highest incidence was observed for lung cancer (4.1% [95% CI: 3.1–5.4]) followed by colorectal cancer (2.4% [95% CI: 2.0–2.7]), gynecological cancer (2.3% [95% CI: 1.6–3.0]), and brain cancer (1.2% [95% CI: 0.7–1.8]) (Fig. 2, Table 2). The cumulative three-year incidence of VTE was 8.8% (95% CI: 8.3–9.3) (Fig. 2, Table 2). The highest cumulative incidence was observed for lung cancer (11.2% [95% CI: 9.4–13.1]), followed by gynecological cancer (9.4% [95% CI: 8.0–10.8]), colorectal cancer (9.0% [95% CI: 8.3–9.7]), and brain cancer (95% CI: 5.4% [4.3–6.6]).

#### Multi-kinase inhibitors

Overall, 3394 patients were treated with MKI, primarily for urogenital (n = 1779; 52%) and hepatobiliary cancer (n = 612; 18%). The median follow-up time was 12.6 months (IQR: 5.2–30.0). During the follow-up period, 111 patients had an ATE (3.3%) and 251 had a VTE (7.4%).

The cumulative three-year incidence of ATE was 3.4% (95% CI: 2.8–4.1). A higher cumulative incidence was observed for urogenital cancer (3.9% [95% CI: 3.1–4.9]) than for hepatobiliary cancer (2.7% [95% CI: 1.6–4.2]) (Fig. 2, Table 2). The three-year cumulative incidence of VTE was 7.5% (95% CI: 6.7–8.5) and similar cumulative incidences were observed in the subgroups of patients with urogenital cancer (7.9% [95% CI: 6.7–9.2]) and hepatobiliary cancer (8.4% [95% CI: 6.4–10.8]) (Fig. 2, Table 2).

#### EGFR-targeted therapy

EGFR-targeted therapies were used in 8603 patients, primarily for colorectal cancer (n = 4166; 48%) and lung cancer (n = 2438; 28%). Over a median follow-up of 11.9 months (5.0–25.0), 216 patients had an ATE (2.5%) and 790 had a VTE (8.6%). The cumulative three-year incidence of ATE was 2.6% (95% CI: 2.2–2.9). The cumulative incidences were 3.3% (95% CI: 2.6–4.0) for lung cancer and 2.6% (95% CI: 2.1–3.1) for colorectal cancer (Fig. 2, Table 2). The three-year cumulative incidence of VTE was 9.3% (95% CI: 8.7–9.9). Again, the cumulative incidences were similar in patients with lung cancer (11.2% [95% CI: 10.0–12.5]) and colorectal cancer (9.2% [95% CI: 8.3–10.1]) (Fig. 2, Table 2).

#### HER2-targeted therapy

Overall, 11,683 patients were treated with HER2-targeted therapies, including 10,370 patients with breast cancer (89%) and 727 with gastroesophageal cancers (6%). A lower incidence of ATE was observed in patients with breast cancer than in patients with gastroesophageal cancer as the three-year cumulative incidences were 1.3% (95% CI: 1.0–1.5) and 2.6% (95% CI: 1.6–4.0), respectively. Furthermore, the cumulative incidence of VTE was 2.9% (95% CI: 2.6–3.3) in patients with breast cancer and 9.2% (95% CI: 7.3–11.5) in patients with gastroesophageal cancers (Supplemental Figure S1).

#### CDK4/6-inhibitors

Overall, 1966 patients were treated with CDK4/6 inhibitors, including 1780 patients with breast cancer. The three-year cumulative incidences of ATE and VTE were 2.6% (95% CI: 1.9–3.5) and 6.9% (95% CI: 5.7–8.3) after initiation of CDK4/6-inhibitors, and 2.3% (95% CI: 1.6–3.2) and 7.1% (95% CI: 5.9–8.5) in the subgroup of patients with breast cancer (Supplemental Figure S1).

#### ALK-/ROS1-targeted therapy

ALK-/ROS1 inhibitors were used in 199 patients, including 180 patients with lung cancer. The three-year cumulative incidences of ATE and VTE were 2.5% (95% CI: 1.0–5.4) and 9.2% (95% CI: 5.7–13.8) overall, and 2.8% (95% CI: 1.1–6.0) and 8.5% (95% CI: 5.0–13.2) in patients with lung cancer.

### Cancer-type cohorts: the association of targeted therapy exposure with ATE and VTE

From all patients diagnosed with cancer during the study period (n = 512,432), we derived distinct cohorts of patients with lung cancer (n = 65,647), colorectal cancer (n = 67,959), melanoma (n = 32,780), urogenital cancers (n = 25,083), breast cancer (n = 75,553), and gynecological cancers (n = 25,894). Patient characteristics of the different cancer-type cohorts are provided in Supplemental Table S8.

In patients with lung cancer (n = 65,647), the HRs for ATE were 0.98 (95% CI: 0.78–1.23) with ICI (n = 3258), 1.22 (95% CI: 0.93–1.59) with EGFR-targeted therapy (n = 2198), 1.57 (95% CI: 1.16–2.13) with VEGF-targeted therapy (n = 1022) and 1.08 (95% CI: 0.45–2.60) with ALK-/ROS-targeted therapy (n = 180). The HRs for VTE were 1.23 with ICI (95% CI: 1.05–1.44), 2.26 with EGFR-targeted therapy (95% CI: 1.94–2.63), 1.58 with VEGF-targeted therapy (95% CI: 1.29–1.95), and 0.83 with ALK-/ROS-targeted therapy (95% CI: 0.45–1.56).

In patients with colorectal cancer (n = 67,959), the HRs for ATE were 1.25 (95% CI: 1.02–1.52) with VEGF-targeted therapy (n = 6769) and 1.38 (95% CI: 1.07–1.79) with EGFR-targeted therapy (n = 3932). The HRs for VTE were 2.31 with VEGF-targeted therapy (95% CI: 2.04–2.60) and 2.47 with EGFR-targeted therapy (95% CI: 2.13–2.85).

In patients with melanoma (n = 32,780), the HR for ATE with ICI therapy (n = 1942) was 1.35 (95% CI: 0.89–2.06) and the HR for VTE was 4.29 (95% CI: 3.10–5.95).

In patients with urogenital cancers (n = 25,083), the HRs for ATE were 1.78 (95% CI: 1.17–2.06) with ICI (n = 1083) and 0.94 (95% CI: 0.65–1.35) with MKI (n = 1715). Furthermore, the HRs for VTE were 1.61 with ICI (95% CI: 1.16–2.23) and 1.49 with MKI (95% CI: 1.16–1.93).

In patients with breast cancer (n = 75,553), the HRs for ATE were 0.81 (95% CI: 0.64–1.04) with HER2-targeted therapy (n = 9246) and 0.73 (95% CI: 0.30–1.76) with CDK4/6-inhibitors (n = 1800). The HRs for VTE were 1.38 with HER2-targeted therapy (95% CI: 1.17–1.62]) and 1.78 with CDK4/6-inhibitors (95% CI: 1.11–2.87).

**Fig. 3:**
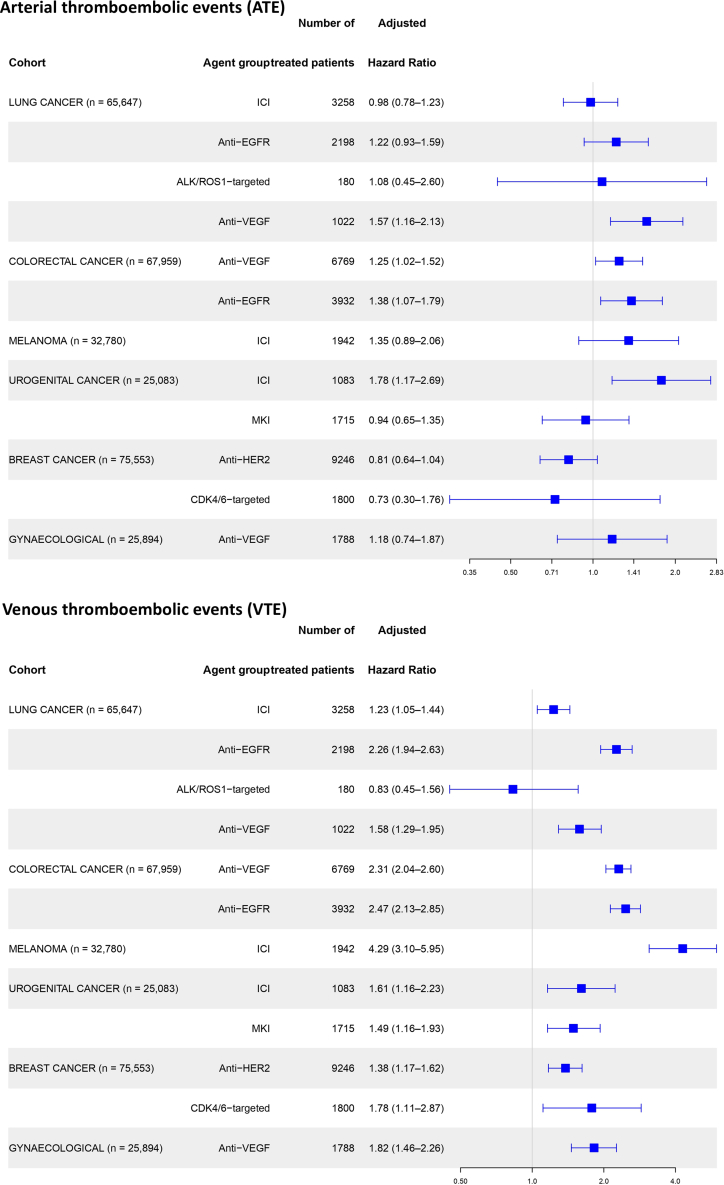
**Cancer-type cohorts: the association between targeted therapy and thromboembolic events.** Adjusted hazard ratios were calculated in multivariable analysis, including patient age at cancer diagnosis, sex, Charlson Comorbidity Index score, cancer stage, and year of cancer diagnosis. Abbreviations: ALK/ROS1: Anaplastic lymphoma kinase or ROS1, CDK4/6: cyclin-dependent kinase 4/6, ICI: immune checkpoint inhibitors, EGFR: epidermal growth factor receptor, HER-2: human epidermal growth factor receptor 2, MKI: multi kinase inhibitors, VEGF: vascular endothelial growth factor.

In patients with gynecological cancers (n = 25,894), the HR for ATE with VEGF-targeted therapy (n = 1788) was 1.18 (95% CI: 0.74–1.87), and the HR for VTE was 1.82 (95% CI: 1.46–2.26). The associations between targeted therapies and ATE and VTE in the different cancer-type cohorts are visualized in Fig. 3, and the results are detailed in Supplemental Table S9.

## Discussion

A clinically relevant risk of ATE and VTE events associated with targeted cancer therapies was observed in this population-based cohort study, varying considerably across targeted therapies and cancer types. No established threshold currently defines a high-risk population for ATE among patients with cancer that affects clinical management.^33^ However, the observed cumulative ATE risk observed in several targeted therapy sub-groups warrants consideration. In contrast, validated risk-stratification tools for VTE are recommended to identify patients with cancer who may benefit from primary prophylactic anticoagulation, including the Khorana score.^34^ Previous studies suggested a 6-month VTE risk of 4–5% in patients with cancer with a Khorana score of ≥2 in the Danish population,^35^^,^^36^ a common threshold for clinically relevant VTE risk. Based on the cumulative risk estimates observed in our study, this threshold is exceeded in several sub-populations treated with targeted therapies.

In detail, our study confirms a clinically relevant risk of thromboembolic events associated with ICI therapy exposure. Recently, published cohort studies have suggested a 2–6% risk of ATE and a 9–25% risk of VTE in ICI-treated patients, which corresponds to the cumulative risks observed in our study.^11^^,^^18^^,^^37^ In a meta-analysis of randomized controlled trials (RCT), the risk of ATE was increased with ICI, whereas the risk of VTE was similar to that in the control patients.^38^ However, these post-hoc analyses are subject to limitations due to potential underreporting of cardiovascular events in clinical trials evaluating ICI.^15^ In various observational studies, a greater risk of ATE and VTE has been observed after ICI therapy compared with that in patients not receiving ICI.^37^^,^^39^, ^40^, ^41^

Furthermore, our findings of an increased thromboembolic risk with VEGF-targeted therapies and MKI are consistent with reports of a potential prothrombotic effect of these anti-angiogenic therapies.^11^ Meta-analyses of RCTs have indicated a 1.4-fold increase in the risk of ATE with VEGF-targeted therapy and a 2–3-fold increase in the risk of ATE with MKI. This corresponds well to the observed risk of ATE associated with MKI and VEGF-targeted therapy in our study.^11^^,^^12^^,^^14^^,^^42^ Conflicting data exist regarding VTE: the largest published meta-analysis reported a 1.3-fold increase in VTE risk with the VEGF-targeted agent bevacizumab,^12^ whereas MKI has not been associated with the risk of VTE.^43^

For EGFR-targeted therapies, the risk of ATE and VTE observed in our study supports previous studies suggesting a modest increase in thromboembolic risk for EGFR-targeted therapies.^44^^,^^45^ A meta-analysis of RCTs reported a 30–40% increase in the risk of ATE and VTE with EGFR-targeted therapies.^45^ In addition, consistent reports from clinical trials evaluating novel EGFR-targeted therapies have indicated a very high VTE risk, thus further supporting the prothrombotic effect of EGFR-targeted therapy.^46^, ^47^, ^48^, ^49^

Recently, targetable alterations in ALK and ROS1 have been identified to promote distinctively prothrombotic cancers, characterized by a risk of VTE as high as 40%.^19^^,^^20^^,^^50^^,^^51^ Interestingly, thromboembolic risk appears to be particularly pronounced at cancer diagnosis, with lower rates observed after the initiation of targeted therapy.^50^^,^^52^ This supports the concept that the underlying tumor biology drives prothrombotic risk, which is further supported by previous data linking ALK-rearrangement to tumoral tissue factor expression.^19^^,^^50^^,^^53^ Consistently, ALK/ROS-targeted therapy exposure was not associated with thromboembolic risk in our study.

Finally, although the risk of thromboembolic events is low in patients with breast cancer,^1^ our findings suggest an elevated risk of VTE in subgroups treated with targeted therapies, especially CDK4/6-inhibitors. Accordingly, in meta-analyses of RCTs, a 2.6-fold increase in VTE risk with CDK4/6-inhibitors has been reported.^21^^,^^54^

Data regarding the underlying mechanisms for thromboembolic events associated with targeted therapies remain scarce. Known risk factors might partly explain the observed incidence of ATE and VTE in treated patients, including cancer type, stage, comorbidities, and often advanced therapeutic setting.^11^ Furthermore, improved cancer-specific survival with targeted therapies prolongs the time-at-risk of thromboembolic events. Moreover, recent studies have identified potential mechanisms that might further contribute to the risk of thromboembolic events associated with selected targeted therapies. For example, a potential pathophysiological link between systemic inflammation and hypercoagulability induced by ICI has been demonstrated in murine studies.^55^^,^^56^ Moreover, ICI has been found to be involved in accelerated atherosclerosis via different pro-inflammatory pathways in murine studies and an increased rate of atherosclerotic plaque progression in humans.^40^^,^^57^^,^^58^ Furthermore, anti-angiogenic therapies have been associated with a prothrombotic effect mediated by impaired fibrinolysis and interference with endothelial integrity.^59^, ^60^, ^61^ In contrast, for other targeted therapies (e.g., EGFR-targeted therapy or CDK4/6 inhibitors), the distinct underlying mechanisms remain elusive. Regarding CDK4/6 inhibitors, somatic mutations in CDK 4 inhibitor B (CDKN2B) were associated with an increased risk of cancer-associated VTE, suggesting a potential pathophysiological link between CDK4/6-inhibiton and thrombotic risk.^62^

Our study has several potential limitations. First, the quality of registry-based data depends on accurate coding of diagnoses and therapies. Danish healthcare registry data have high validity regarding coding of cardiovascular outcomes, with positive predictive values (PPV) of 88% for VTE and 97%, 88%, and 91% for coding of myocardial infarction, ischemic stroke, and peripheral arterial occlusion, respectively.^23^^,^^63^ Additionally, the validity of data on systemic cancer therapies in the DNPR (PPV: 92%) and conditions included in the Charlson Comorbidity Index (PPV: 95%) is high.^26^^,^^30^ Second, several important variables were not available in the registries, including body mass index and smoking status. Therefore, residual confounding by unknown variables cannot be ruled out. Third, the analysis of thromboembolic risk according to exposure to targeted therapy was partly limited by small sample size in some subgroups. Similarly, because of the relatively low numbers of ATE outcomes, these analyses might have been affected by limited statistical precision. Fourth, data on individual agents and concomitant cancer therapies (e.g., chemotherapy, surgery, radiotherapy, and targeted therapy combinations) were not analyzed because of sample size limitations. Therefore, the baseline risk of ATE and VTE within targeted therapy cohorts might be affected by concomitant cancer therapies. Furthermore, results from Cox proportional hazards regression analyses in cancer-type cohorts should be considered exploratory and hypothesis-generating. Lastly, in the time-varying analyses of the cancer-type cohorts, the unexposed cancer cohorts may include heterogeneous populations, including patients who were not eligible for targeted therapy. To address this limitation and reduce confounding by indication, we conducted the time-dependent analyses within selected cancer-type cohorts and adjusted the hazard ratios for eligibility-related covariates, including cancer stage, age, sex, Charlson Comorbidity Index score, and year of cancer diagnosis. However, residual confounding by indication—where the clinical factors influencing the decision to initiate targeted therapy are also associated with the outcome—cannot be ruled out.

Our study has important clinical implications. The substantial but heterogeneous risk of thromboembolic events observed with exposure to targeted therapy underscores the need for improved and treatment-specific cardiovascular surveillance and prevention strategies. Therefore, future studies should focus on identifying risk factors and biomarkers for predicting thromboembolic events in patients receiving targeted cancer therapy. Furthermore, given the underreporting of thromboembolic events in clinical trials investigating targeted cancer therapies, consistent reporting of cardiovascular events should be prioritized. Finally, dedicated studies should focus on identifying potential mechanisms of cardiovascular toxicity in targeted cancer therapies.

## Contributors

Florian Moik contributed to study design, data analysis, data interpretation, and manuscript writing. Erzsébet Horváth-Puhó contributed to study design, data analysis, data interpretation, and manuscript writing. Cihan Ay contributed to study design, data interpretation, and manuscript writing. Ingrid Pabinger contributed to data interpretation and manuscript writing. Frits Mulder contributed to data interpretation and manuscript writing. Nick van Es contributed to study design, data interpretation, and manuscript writing. Henrik Toft Sørensen contributed to study design, data analysis, data interpretation, and manuscript writing. All authors read and approved the final version of the manuscript. Erzsébet Horváth-Puhó and Henrik Toft Sørensen had access to and verified the underlying data.

## Data sharing statement

The registry data used in this study are considered personal and protected patient data, in accordance with the Danish Data Protection act and the General Data Protection Regulations, and therefore cannot be disclosed. Data and material will not be available due to Danish Data Legislation. Access to raw data can be requested through the Danish Data Health Agency.

## Declaration of interests

All authors declare that there are no conflicts of interest associated with the manuscript.
