# Supplementary material for: Arterial and venous thromboembolic events in patients with cancer treated with targeted therapies: a population-based cohort study
Source: eClinicalMedicine. 2025 Aug 21;87:103440. doi: 10.1016/j.eclinm.2025.103440 (PMC12396447; doi:10.1016/j.eclinm.2025.103440)
Supplement: Supplemental Tables and Figure [file mmc1.docx]

**Supplemental Data**

[**Supplemental Data** 1](#_Toc200213832)

[Supplemental Table 1. Description of the used Danish registries 3](#_Toc200213833)

[Supplemental Table 2. Targeted cancer therapy codes used in the study 4](#_Toc200213842)

[Supplemental Table 3. Diagnosis codes used in the study 5](#_Toc200213843)

[Supplemental Table 5. Targeted therapy cohorts: Duration of targeted cancer therapies 7](#_Toc200213844)

[Supplemental Table 6. Targeted therapy cohorts: Number of outcome events over three-year follow-up period after initiation of targeted therapy 8](#_Toc200213845)

[Supplemental Table 7. Between-group overlap between different targeted therapy cohorts. 9](#_Toc200213846)

[Supplemental Table 8. Cancer-type cohorts: patient characteristics 10](#_Toc200213847)

[Supplemental Table 9. Cancer-type cohorts: adjusted hazard ratios of ATE and VTE 11](#_Toc200213848)

[Supplemental Figure 1: Targeted therapy cohorts: Cumulative risk of VTE and ATE in HER2- and CDK4/6-targeted therapies 12](#_Toc200213849)

**Supplemental Table 1. Description of the used Danish registries**

| **Danish Cancer Registry** | The Danish Cancer Registry has recorded all incident cases of malignant neoplasms in Denmark since 1943, including information on morphology, histology, and cancer stage at time of diagnosis. Cancer cases are coded according to the *International Classification of Diseases, Tenth Revision*. We used this Registry to identify all patients with a new cancer diagnosis during the study period. |
| --- | --- |
| **Danish Civil Registration System** | The Danish Civil Registration System, established in 1968, is updated daily to reflect changes in vital status and migration for the entire population of Denmark. Each resident is assigned a unique ten-digit personal identification number at birth or upon immigration, allowing unambiguous linkage of individual data across all data sources. |
| **Danish National Patient Registry** | This Registry contains information on hospital inpatient admissions since 1977 and hospital outpatient clinic and emergency contacts since 1995. Each hospital discharge or outpatient visit is recorded with one primary diagnosis and one or more secondary diagnoses coded according to the *International Classification of Diseases, Eighth Revision* until 1993 and *Tenth Revision* thereafter. We used the Danish National Patient Registry to identify the patients treated with targeted cancer therapies, define the outcome events and comorbidities included in the Charlson Comorbidity Index. |
| **Danish National Prescription Registry** | Since 1995, the Prescription Registry has collected comprehensive data on all prescription drugs sold in Danish pharmacies. The registry includes variables that describe each filled prescription, such as information about the patient, the dispensed drug, the prescribing healthcare provider, and the dispensing pharmacy. Prescription drugs are coded using the *Anatomical Therapeutic Chemical* (ATC) coding system. Targeted cancer therapies were identified also from the prescription drugs dispensed in Danish pharmacies. |

**Supplemental Table 2. Targeted cancer therapy codes used in the study**

| **Targeted therapy** | **Individual agents (Target)** | **Procedure codes** | **ATC codes** |
| --- | --- | --- | --- |
| **Immune-checkpoint-inhibitors** | Anti-PD-L1-treatment | **BOHJ19J** |  |
|  | Anti-CTLA4 treatment | **BOHJ19D** |  |
|  | Pembrolizumab  (PD-1) | **BOHJ19J3** | **L01XC18** |
|  | Nivolumab (PD-1) | **BOHJ19H2** | **L01XC17** |
|  | Atezolizumab (PD-L1) | **BOHJ19J2** | **L01XC32** |
|  | Durvalumab (PD-L1) | **BOHJ19H7** | **L01XC28** |
|  | Ipililumab (CTLA4) | **BOHJ19D1** | **L01XC28** |
|  | Avelumab (PD-L1) | **BOHJ19J1** | **L01XC31** |
|  | Cemiplimab (PD-1) | **BOHJ19J4** | **L01XC33** |
| **Anti-VEGF** | Anti-vascular endothelial growth factor (anti-VEGF) | **BOHJ19B** |  |
|  | Bevacizumab | **BOHJ19B1** | **L01XC07** |
|  | Ramucirumab | **BOHJ19B2** | **L01XC21** |
| **Multi-kinase-inhibitors** | Sorafenib | **BWHA407** | **L01XE05** |
|  | Sunitinib | **BWHA406** | **L01XE04** |
|  | Pazopanib | **BWHA410** | **L01XE11** |
|  | Vandetanib |  | **L01XE12** |
|  | Axitinib | **BWHA426** | **L01XE17** |
|  | Regorafenib | **BWHA422** | **L01XE21** |
|  | Cabozantinib | **BWHA424** | **L01XE26** |
|  | Lenvatinib |  | **L01XE29** |
|  | Nintedanib | **BWHA449** | **L01XE31** |
|  | Tivozanib | **BWHA436** | **L01XE34** |
| **Anti-EGFR** | Anti-epidermal growth factor receptor (anti-EGFR) | **BOHJ19C** |  |
|  | Gefitinib |  | **L01XE02** |
|  | Erlotinib | **BWHA404** | **L01XE03** |
|  | Lapatinib | **BWHA405** | **L01XE07** |
|  | Cetixumab | **BOHJ17** | **L01XC06** |
|  | Osimertinib | **BWHA434** | **L01XE35** |
|  | Panitumumab | **BOHJ19C2** | **L01XC08** |
|  | Necitumumab |  | **L01XC22** |
|  | Olmutinib |  | **L01XE40** |
|  | Afatinib | **BWHA417** | **L01XE13** |
|  | Brigatinib |  | **L01XE43** |
|  | Dacomitinib |  | **L01XE47** |
|  | Icotinib |  | **L01XE48** |
| **HER2/neu** | Trastuzumab | **BOHJ13** | **L01XC03** |
|  | Pertuzumab | **BOHJ19H3** | **L01XC13** |
|  | Trastuzumab emtansine | **BOHJ13A** | **L01XC14** |
|  | Neratinib | **BWHA414** | **L01XE45** |
| **ALK/ROS** | Crizotinib | **BWHA413** | **L01XE16** |
|  | Ceritinib | **BWHA431** | **L01XE28** |
|  | Alectinib | **BWHA440** | **L01XE36** |
|  | Brigatinib |  | **L01XE43** |
|  | Lorlatinib | **BWHA448** | **L01XE44** |
| **CKD4/6 inhibitors** |  |  | **ML01EF** |
|  | Palbociclib | **BWHA442** | **L01EF01** |
|  | Abemaciclib | **BWHA444** | **L01EF03** |
|  | Ribociclib | **BWHA430** | **L01EF02** |

**Supplemental Table 3. Diagnosis codes used in the study**

|  | **ICD-10 codes** | **ICD-8 codes** |
| --- | --- | --- |
| **Cancer** | C00-97 (except C44) |  |
| **ATE** |  |  |
| Myocardial infarction | I21 | 410 |
| Peripheral arterial occlusion | I74 | 444 |
| Ischemic stroke | I63 | 433, 434 |
| Unspecified stroke | I64 |  |
| **VTE** |  |  |
| Pulmonary embolism | I26 | 450 |
| Deep vein thrombosis | I801-803 | 451 |
| Other VTE | I808, I809, I81, I82, O882, I676 | 452-453, 671, 321 |
|  |  |  |
| **Charlson Comorbidity Index** |  |  |
| Myocardial infarction | I21; I22; I23 | 410 |
| Congestive heart failure | I50; I11.0; I13.0; I13.2 | 427.09; 427.10; 427.11; 427.19; 428.99; 782.49 |
| Peripheral vascular disease | I70; I71; I72; I73; I74; I77 | 440; 441; 442; 443; 444; 445 |
| Cerebrovascular disease | I60-I69; G45; G46 | 430-438 |
| Chronic pulmonary disease (COPD) | J40-J47; J60-J67; J68.4; J70.1;  J70.3; J84.1; J92.0; J96.1; J98.2; J98.3 | 490-493; 515-518 |
| Rheumatic/Connective tissue disease | M05; M06; M08; M09; M30;M31;  M32; M33; M34; M35; M36; D86 | 712; 716; 734; 446; 135.99 |
| Peptic ulcer disease | K22.1; K25-K28 | 530.91; 530.98; 531-534 |
| Liver disease |  |  |
| Mild | B18; K70.0-K70.3; K70.9; K71; K73; K74; K76.0 | 571; 573.01; 573.04 |
| Moderate to severe | B15.0; B16.0; B16.2; B19.0; K70.4; K72; K76.6; I85 | 070.00; 070.02; 070.04; 070.06; 070.08; 573.00; 456.00-456.09 |
| Diabetes |  |  |
| Uncomplicated | E10.0, E10.1; E10.9  E11.0; E11.1; E11.9 | 249.00; 249.06; 249.07; 249.09  250.00; 250.06; 250.07; 250.09 |
| With end organ damage | E10.2-E10.8  E11.2-E11.8 | 249.01-249.05; 249.08  250.01-250.05; 250.08 |
| Hemiplegia | G81; G82 | 344 |
| Moderate to severe renal disease | I12; I13; N00-N05; N07; N11; N14; N17-N19; Q61 | 403; 404; 580-583; 584; 590.09; 593.19; 753.10-753.19; 792 |
| AIDS | B21-B24 | 079.83 |

**Abbreviations**: AIDS: Acquired immunodeficiency syndrome; ATE: Arterial thromboembolic events, ICD: International Classification of Diseases; VTE: Venous thromboembolism;

**Supplemental Table 4. Targeted therapy cohorts: Cancer types of treated patients**

|  | **Targeted therapy (n = 41,744)** | **ICI (n = 7,880)** | **VEGF-targeted (n = 12,802)** | **MKI (n = 3,394)** | **Her2- targeted (n = 11,683)** | **EGFR-targeted (n = 8,603)** | **ALK/ROS-targeted (n = 199)** | **CDK4/6-targeted**  **(n = 1,966)** |
| --- | --- | --- | --- | --- | --- | --- | --- | --- |
| **Gastro-esophageal** | 1,243 (3.0%) | 70 (0.9%) | 233 (1.8%) | 40 (1.2%) | 727 (6.2%) | 273 (3.2%) | - | - |
| **CRC** | 9,355 (22.4%) | 179 (2.3%) | 6,983 (54.5%) | 171 (5.0%) | 168 (1.4%) | 4,166 (48.4%) | <5 | 25 (1.3%) |
| **Hepatobiliary** | 923 (2.2%) | 65 (0.8%) | 195 (1.5%) | 612 (18.0%) | <5 | 187 (2.2%) | - | - |
| **Pancreatic** | 271 (0.6%) | 105 (1.3%) | 58 (0.5%) | 32 (1.0%) | 16 (0.1%) | 67 (0.8%) | - | <5 |
| **Lung** | 6,716 (16.1%) | 3368 (42.7%) | 1,141 (8.9%) | 51 (1.5%) | 91 (0.8%) | 2,438 (28.3%) | 180 (90.5%) | 24 (1.2%) |
| **Breast** | 12,389 (29.7%) | 154 (2.0%) | 189 (1.5%) | 74 (2.2%) | 10,370 (88.8%) | 481 (5.6%) | <5 | 1,780 (90.5%) |
| **Gynecologic** | 1,927 (4.6%) | 85 (1.1%) | 1,783 (13.9%) | 47 (1.4%) | 49 (0.4%) | 43 (0.5%) | - | 14 (0.7%) |
| **Prostate** | 273 (0.7%) | 121 (1.5%) | 63 (0.5%) | 41 (1.2%) | 19 (0.2%) | 45 (0.5%) | <5 | <5 |
| **Urogenital** | 2,412 (5.8%) | 917 (11.6%) | 137 (1.1%) | 1,779 (52.4%) | 21 (0.2%) | 31 (0.4%) | - | 6 (0.3%) |
| **Brain** | 1,482 (3.6%) | 62 (0.8%) | 1,434 (11.2%) | 6 (0.2%) | <15 | 96 (1.1%) | - | - |
| **Hematologic** | 380 (0.9%) | 154 (2.0%) | 24 (0.2%) | 46 (1.4%) | 35 (0.3%) | 31 (0.4%) | <5 | 7 (0.4%) |
| **Melanoma** | 1,928 (4.6%) | 1,855 (23.5%) | 27 (0.2%) | 28 (0.8%) | 15 (0.1%) | 15 (0.2%) | <5 | 9 (0.5%) |
| **Others** | 2,445 (5.9%) | 745 (9.5%) | 535 (4.2%) | 465 (13.7%) | 157 (1.3%) | 730 (8.5%) | 10 (5.0%) | 96 (4.9%) |

Values indicate count and percentages (%). Abbreviations: ALK: anaplastic lymphoma kinase; CDK: cyclin dependent kinase; CRC: colorectal cancer; EGFR: epidermal growth factor receptor; HER2: human epidermal growth factor receptor 2; ICI: immune checkpoint inhibitors; MKI: multi-kinase inhibitors; VEGF: vascular endothelial growth factor.

**Supplemental Table 5. Targeted therapy cohorts: Duration of targeted cancer therapies**

| **Targeted therapy cohorts** | **n** | **Median time on treatment in months (IQR)** |
| --- | --- | --- |
| Targeted therapies | 41,744 | 9 (4-15) |
| Immune checkpoint Inhibitors (ICI) | 7,880 | 6 (4-11) |
| Anti-angiogenic (VEGF-targeted) | 12,802 | 7 (4-12) |
| Multi-kinase inhibitors (MKI) | 3,394 | 6 (3-15) |
| EGFR-targeted | 8,603 | 5 (3-10) |
| HER2-targeted | 11,683 | 14 (10-15) |
| CDK4/6 inhibitors | 1,966 | 9 (5-18) |
| ALK/ROS-targeted | 199 | 7 (3-18) |

**Abbreviations**: ALK: anaplastic lymphoma kinase; CDK: cyclin dependent kinase; EGFR: epidermal growth factor receptor; HER2: human epidermal growth factor receptor 2; ICI: immune checkpoint inhibitors; IQR: interquartile range; MKI: multi-kinase inhibitors; VEGF: vascular endothelial growth factor.

**Supplemental Table 6. Targeted therapy cohorts: Number of outcome events over three-year follow-up period after initiation of targeted therapy**

|  | **Targeted therapy (n = 41,744)** | **ICI (n = 7,880)** | **VEGF-targeted (n = 12,802)** | **MKI (n = 3,394)** | **Her2- targeted (n = 11,683)** | **EGFR-targeted (n = 8,603)** | **ALK/ROS-targeted (n = 199)** | **CDK4/6-targeted**  **(n = 1,966)** |
| --- | --- | --- | --- | --- | --- | --- | --- | --- |
| **VTE** | 2,908 (7.0%) | 591 (7.5%) | 1,104 (8.6%) | 251 (7.4%) | 388 (3.3%) | 790 (9.2%) | 18 (9.0%) | 117 (6.0%) |
| PE | 1,726 (4.1%) | 396 (5.0%) | 624 (4.9%) | 128 (3.8%) | 211 (1.8%) | 477 (5.5%) | n/a | n/a |
| DVT | 606 (1.5%) | 87 (1.1%) | 288 (2.2%) | 40 (1.2%) | 87 (0.7%) | 154 (1.8%) | n/a | n/a |
| Other VTE | 576 (1.4%) | 108 (1.4%) | 192 (1.5%) | 83 (2.4%) | 90 (0.8%) | 159 (1.8%) | n/a | n/a |
| **ATE** | 979 (2.3%) | 245 (3.1%) | 297 (2.3%) | 111 (3.3%) | 151 (1.3%) | 216 (2.5%) | n/a | n/a |
| MI | 312 (0.7%) | 82 (1.0%) | 79 (0.6%) | n/a | 52 (0.4%) | 66 (0.8%) | n/a | n/a |
| PAO | 66 (0.2%) | 11 (0.1%) | 27 (0.2%) | n/a | 10 (0.1%) | 24 (0.3%) | n/a | n/a |
| Ischemic Stroke | 601 (1.4%) | 152 (2.0%) | 191 (1.5%) | 56 (1.6%) | 89 (0.7%) | 126 (1.5%) | n/a | n/a |

Data not reported if a subgroup for ATE and/or VTE included ≤5 patients. Abbreviations: ALK: anaplastic lymphoma kinase; ATE: arterial thromboembolic events; CDK: cyclin dependent kinase; DVT: deep vein thrombosis; EGFR: epidermal growth factor receptor; HER2: human epidermal growth factor receptor 2; ICI: immune checkpoint inhibitors; MKI: multi-kinase inhibitors; MI: myocardial infarction; PAO: peripheral arterial occlusion; PE: pulmonary embolism; VEGF: vascular endothelial growth factor; VTE: venous thromboembolism.

## Supplemental Table 7. Between-group overlap between different targeted therapy cohorts.

|  | **ICI (n = 7,880)** | **VEGF-targeted (n = 12,802)** | **MKI (n = 3,394)** | **EGFR-targeted (n = 8,603)** | **Her2- targeted (n = 11,683)** | **CDK4/6-targeted (n = 1,966)** | **ALK/ROS-targeted (n = 199)** |
| --- | --- | --- | --- | --- | --- | --- | --- |
| **ICI (n = 7,880)** | n/a | 211 (2.7%) | 455 (5.8%) | 210 (2.7%) | 64 (0.8%) | <10 (<0.1%) | 24 (0.3%) |
| **VEGF-targeted (n = 12,802)** | 211 (1.6%) | n/a | 201 (1.6%) | 2650 (20.7%) | 261 (2.0%) | <10 (<0.1%) | <10 (<0.1%) |
| **MKI (n = 3,394)** | 455 (13.4%) | 201 (5.9%) | n/a | 84 (2.5%) | <10 (<0.3%) | 42 (1.2%) | <10 (<0.3%) |
| **EGFR-targeted (n = 8,603)** | 210 (2.4%) | 2650 (30.8%) | 84 (1.0%) | n/a | 550 (6.4%) | 10 (0.1%) | 26 (0.3%) |
| **Her2- targeted (n = 11,683)** | 64 (0.5%) | 261 (2.2%) | <10 (<0.1%) | 550 (4.7%) | n/a | 112 (1.0%) | <10 (<0.1%) |
| **CDK4/6-targeted (n = 1,966)** | <10 (<0.5%) | <10 (<0.5%) | 42 (2.1%) | 10 (0.5%) | 112 (5.7%) | n/a | <10 (<0.5%) |
| **ALK/ROS-targeted (n = 199)** | 24 (12.1%) | <10 (5.0%) | <10 (5.0%) | 26 (13.1%) | <10 (5.0%) | <10 (5.0%) | n/a |

**Table legend:** Percentages calculated using row totals as denominators. ALK: anaplastic lymphoma kinase; CDK: cyclin dependent kinase; EGFR: epidermal growth factor receptor; HER2: human epidermal growth factor receptor 2; ICI: immune checkpoint inhibitors; MKI: multi-kinase inhibitors; VEGF: vascular endothelial growth factor

**Supplemental Table 8. Cancer-type cohorts: patient characteristics**

| **Cancer type** | **Colorectal cancer (n = 67,959)** | **Lung cancer (n = 65,647)** | **Breast cancer (n = 75,553)** | **Melanoma (n = 32,780)** | **Gynaecologic cancer (n = 25,894)** | **Urogenital cancer (n = 25,083)** |
| --- | --- | --- | --- | --- | --- | --- |
| **Age*** | 71 (63-79) | 70 (63-77) | 64 (53-73) | 60 (47-72) | 65 (54-74) | 70 (62-78) |
| **Female** | 31,480 (46%) | 32,021 (49%) | 75,058 (99%) | 17,649 (54%) | 25,894 (100%) | 7,543 (30%) |
| **Cancer stage*** |  |  |  |  |  |  |
| **- Local** | 8,247 (12.1%) | 8,473 (12.9%) | 39,289 (52.0%) | 20,213 (61.7%) | 10,533 (40.7%) | 6,011 (24.0%) |
| **- Regional** | 30,274 (44.5%) | 17,302 (26.4%) | 22,158 (29.3%) | 4,866 (14.8%) | 5,754 (22.2%) | 6,092 (24.3%) |
| **- Distant** | 14,258 (21%) | 33,400 (51%) | 2,633 (4%) | 908 (3%) | 3,933 (15%) | 4,072 (16%) |
| **CCI*** |  |  |  |  |  |  |
| **- 0** | 42,773 (63%) | 31,686 (48%) | 56,700 (75%) | 25,351 (77%) | 18,906 (73%) | 14,073 (56%) |
| **- 1** | 14,472 (21%) | 17,978 (27%) | 12,273 (16%) | 4,696 (14%) | 4,433 (17%) | 5,687 (23%) |
| **- 2+** | 10,714 (16%) | 15,983 (24%) | 6,580 (9%) | 2,733 (8%) | 2,555 (10%) | 5,323 (21%) |
| **Year of cancer diagnosis** |  |  |  |  |  |  |
| **2004-2009** | 22,096 (33%) | 22,247 (34%) | 25,765 (34%) | 8,844 (27%) | 9,048 (35%) | 8,066 (32%) |
| **2010-2015** | 24,946 (37%) | 23,400 (36%) | 21,176 (36%) | 12,016 (37%) | 9338 (36%) | 9,057 (36%) |
| **2016-2020** | 20,917 (31%) | 20,000 (31%) | 22,612 (30%) | 11,920 (36%) | 7,508 (29%) | 7,960 (32%) |
| **Targeted therapy use** | 9,074 (13%) | 6,251 (10%) | 11,577 (15%) | 2,142 (7%) | 2,027 (8%) | 2,435 (10%) |
| **Median time to targeted therapy use (years)** | 0.7 (0.1-2.1) | 0.4 (0.1-1.2) | 0.4 (0.3-1.1) | 1.9 (0.3-4.8) | 1.1 (0.4-3.1) | 0.7 (0.2-2.5) |
| **Time to targeted therapy <12 months** | 5039 (56%) | 4407 (71%) | 8612 (74%) | 791 (37%) | 988 (49%) | 1415 (58%) |
| **Median follow-up (years)**** | 3.0 (1.2-3.0) | 0.7 (0.2-1.9) | 3.0 (3.0-3.0) | 3.0 (1.7-3.0) | 2.8 (0.9-3.0) | 3.0 (3.0-3.0) |

*At cancer diagnosis

**Defined as time to death, emigration, or a maximum of 3 years.

Values indicate median and interquartile range (IQR), or count and percentages (%).

**Supplemental Table 9. Cancer-type cohorts: adjusted hazard ratios of ATE and VTE**

| **Cancer type** | **Targeted therapy** | **HR for ATE (95% CI)*** | **HR for VTE (95% CI)*** |
| --- | --- | --- | --- |
| **Lung cancer** (n = 65,647) | **ICI** (n = 3,258) | 0.98 (0.78–1.23) | 1.23 (1.05–1.44) |
|  | **Anti-EGFR** (n = 2,198) | 1.22 (0.93–1.59) | 2.26 (1.94–2.63) |
|  | **ALK/ROS-targeted** (n = 180) | 1.08 (0.45–2.60) | 1.58 (1.29–1.95) |
|  | **Anti-VEGF** (n = 1,022) | 1.57 (1.16–2.13) | 0.83 (0.45–1.56) |
| **Colorectal cancer**  (n = 67,959) | **Anti-VEGF** (n = 6,769) | 1.25 (1.02–1.52) | 2.31 (2.04–2.60) |
|  | **Anti-EGFR** (n = 3,932) | 1.38 (1.07–1.79) | 2.47 (2.13–2.85) |
| **Melanoma**  (n = 32,780) | **ICI** (n = 1,942) | 1.35 (0.89–2.06) | 4.29 (3.10–5.95) |
| **Urogenital cancer**  (n = 25,083) | **ICI** (n = 1,083) | 1.78 (1.17–2.69) | 1.61 (1.16–2.23) |
|  | **MKI** (n = 1,715) | 0.94 (0.65–1.35) | 1.49 (1.16–1.93) |
| **Breast cancer**  (n = 75,553) | **Anti-HER2** (n = 9,246) | 0.81 (0.64–1.04) | 1.38 (1.17–1.62) |
|  | **CDK4/6-targeted** (n = 1,800) | 0.73 (0.30–1.76) | 1.78 (1.11–2.87) |
| **Gynecological cancer** (n = 25,894) | **Anti-VEGF** (n = 1,788) | 1.18 (0.74–1.87) | 1.82 (1.46–2.26) |

*Exposure to the specified targeted agent was treated as time-dependent variable in a Cox proportional hazards regression model. Multivariable adjustment for age, sex, Charlson comorbidity index, cancer stage, and year of cancer diagnosis.

Abbreviations: ALK: anaplastic lymphoma kinase; ATE: arterial thromboembolic events; CDK: cyclin dependent kinase; CI: confidence interval; EGFR: epidermal growth factor receptor; HER2: human epidermal growth factor receptor 2; HR: hazard ratio; ICI: immune checkpoint inhibitors; MKI: multi-kinase inhibitors; VEGF: vascular endothelial growth factor; VTE: venous thromboembolism.

**Supplemental Figure 1: Targeted therapy cohorts: Cumulative risk of VTE and ATE in HER2- and CDK4/6-targeted therapies**

1. **ATE**


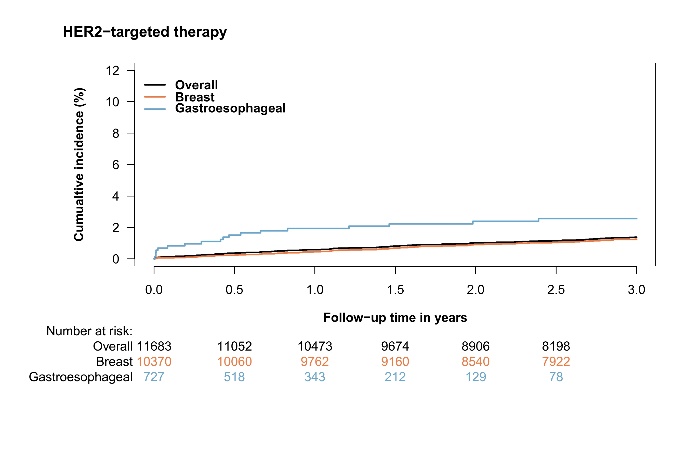

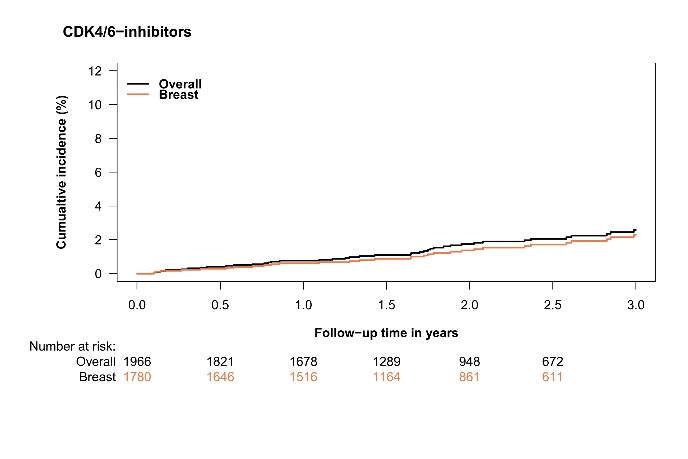


1. **VTE**


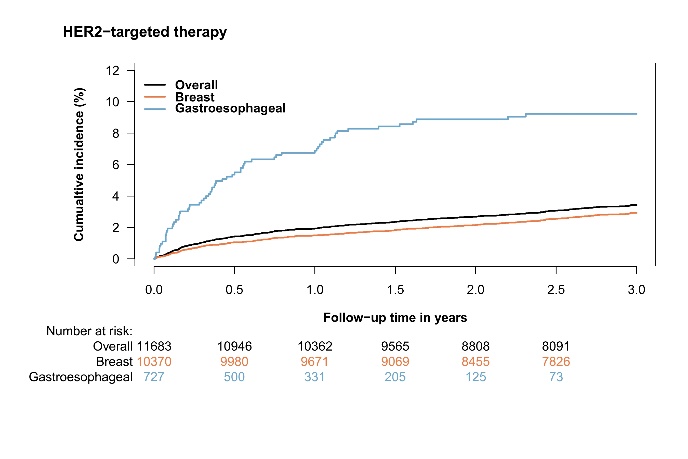

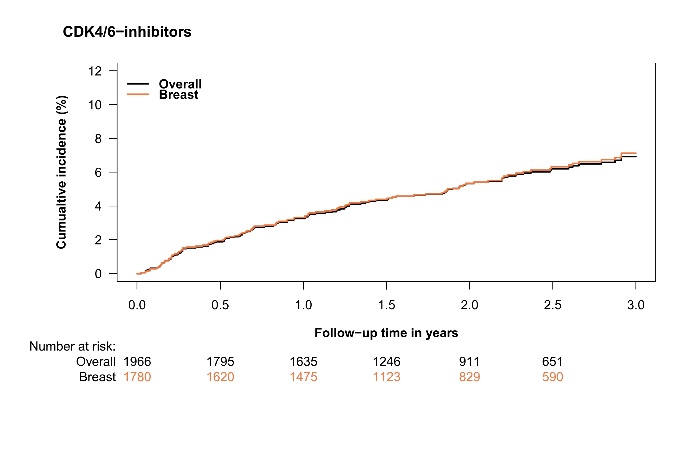


**Figure legend**: Abbreviation: ATE: arterial thromboembolic event, CDK4/6: cyclin‐dependent kinase 4/6, HER-2: human epidermal growth factor receptor 2, VTE: venous thromboembolism.
